# The impact of COVID-19 pandemic on pet behavior and human-animal interaction: a longitudinal survey-based study in the United States

**DOI:** 10.3389/fvets.2023.1291703

**Published:** 2023-11-28

**Authors:** Hsin-Yi Weng, Niwako Ogata

**Affiliations:** ^1^Department of Comparative Pathobiology, College of Veterinary Medicine, West Lafayette, IN, United States; ^2^Department of Veterinary Clinical Sciences, College of Veterinary Medicine, West Lafayette, IN, United States

**Keywords:** dogs, cats, companion animal, behavior problems, COVID-19, human-animal interaction

## Abstract

**Introduction:**

Although multiple studies have explored behavioral changes in pets during the strict lockdown of the COVID-19 pandemic between May and July 2020, this longitudinal study uniquely investigated the phases beyond strict lockdown. The primary objective of this research was to evaluate the pandemic’s impact on pet behavior.

**Methods:**

To achieve this goal, we conducted an online survey, completed by pet owners residing in the United States between June 2020 (including retrospective data for February and April 2020) and December 2021. The study encompassed four distinct pandemic phases: Pre-pandemic (data collected retrospectively), Strict lockdown, Prolonged lockdown, and Re-opening.

**Results and discussion:**

The data collected from surveys completed by 3,278 pet owners across one to six time points revealed declining trends in all investigated behavior problems of both dogs and cats. Concurrently, human-animal interaction activities also showed variations across different COVID-19 phases. The association between human-animal interactions and the occurrence of behavior problems underscored the importance of not only exercise quantity but also adherence to consistent schedules, particularly for dogs, in mitigating behavior problems. Interestingly, among cats, sleeping outside the bedroom was associated with a reduced occurrence of aggression, anxiety, fear, and physiological behavior changes. However, sleep location displayed limited or no association with behavior problems in dogs. In summary, this study highlights the importance of tailoring interventions to the unique needs of each species through human-animal interactions to mitigate the occurrence of behavioral problems and enhance human-animal relationships.

## Introduction

The activity level and behavior of pets have long been acknowledged to be greatly influenced by the lifestyle patterns of their owners (1, 2). The abrupt and dramatic changes in the lifestyle patterns of pet owners due to the COVID-19 pandemic have raised concerns about the potential impact on the physiological and behavioral health of pets.

To investigate the impact of the COVID-19 pandemic on changes in dog and cat behavior several studies were conducted (3–8). For example, Jezierski et al. conducted in primarily European countries between May 1st and June 30th, 2020 (9, 10), during which lockdown and stay-at-home orders were implemented in many of these countries. The study findings revealed that the increased time spent at home during the pandemic led to more frequent engagement in activities such as increased dog walking and dedicating additional time to playing and petting their animals. Dog owners reported overall positive behavior changes, such as increased calmness or playfulness in their dogs. However, owners who underwent lockdown were 1.8 times more likely to report certain negative behavior changes in their dogs as well, such as more frequent barking or anxiety (9). On the other hand, cat owners primarily reported no behavior changes in their cats (10). In cases where 33% of owners reported cat behavior changes, they were mostly positive behavior changes (10).

Nevertheless, the surge in the number of household members, including children, staying at home for a longer period of time and the increased engagement in home-based activities could also lead to new challenges (5). Interviews conducted with 15 owners of 18 dogs between December 2021 and January 2022 revealed that many noticed changes in their dogs’ behaviors during or after the lockdown period. These changes included increases in fear-related and aggressive behaviors. Notably, other studies reported an increase in incidents involving children being bitten by dogs during the pandemic in 2020, compared to previous years (11, 12). It is not surprising that the overall behavior and activity of household pets are mostly influenced by the presence and absence of owners and other household members at home (2). For example, cats that were accustomed to having less stimulation during the day when humans were absent before the COVID-19 pandemic might experience disruptions in their natural diurnal rhythms with the continuous presence of family members at home.

The pandemic also led to an increase in the number of new pet-owning households, with a study in the United Kingdom reporting a peak between March and December 2020 (13). While a peak in new pet ownership was observed in spring 2020 based on Google Trends data from various countries, including Australia, the United States, Canada, New Zealand, the United Kingdom, Singapore, the Philippines, and Malaysia, this trend diminished after July 2020, especially in dogs (14). Individuals obtaining puppies during the pandemic exhibited characteristics different from those who acquired puppies before the pandemic (i.e., 2019), being less likely to have prior dog ownership experience, potentially posing risks for the future well-being of these puppies (13). Moreover, there was an alarming increase in the number of people giving up their pets and the number of new families obtaining pets in the United States, including Hawaii and Alaska, during the pandemic (15).

The pandemic also had a disruptive effect on the socialization and training opportunities for newly adopted pets, especially puppies and kittens. With the implementation of lockdown measures, many puppy socialization classes and training schools shifted to online platforms, limiting opportunities for interaction with strangers and neighbor’s pets (16). A study by Sacchettino et al. (17) found that dogs that underwent their socialization period during the lockdown experienced a significant increase in fear and aggression later in life. Furthermore, concerns have been raised about the possibility of pets experiencing new or recurrent behavior problems when their owners transitioned from working at home to returning to the workplace (14).

Most studies investigated behavior changes in pets collected data only during the COVID lockdown period between May and July 2020. While human activities were mostly restricted during that period, these cross-sectional studies were limited in investigating the dynamic changes of human-pet interactions and their influences on pet behavior throughout the pandemic. To fill the knowledge gaps, we conducted a survey-based longitudinal study on pet owners in the US, spanning through pre-, during-, and late-pandemic phases from February 2020 to December 2021. The study aimed to investigate the impact of COVID-19 on pet behavior problems and human-animal interactions. Our hypotheses were as follows: (1) the occurrence of behavior problems in pet dogs and cats would vary across different phases of the COVID-19 pandemic, (2) human-animal interaction (HAI) activities would also vary across COVID phases, and (3) there would be an association between HAI and pet behavior during the COVID-19 pandemic.

By examining these hypotheses, we sought to gain valuable insights into the effects of the COVID-19 pandemic on pet behavior and human-animal relationships, which would inform strategies to address potential challenges and promote positive interactions between pets and their owners during times of crisis.

## Materials and methods

### Recruitment

The study protocol was approved by the Purdue University Human Research Protection Program Institutional Review Board (IRB-202-760). Two online surveys, one for dog owners and the other for cat owners, were developed using Qualtrics (Qualtrics Inc., Provo, UT). Participants were recruited via an online crowdsourcing platform (CloudResearch^®^ Prime Research Solutions LLC) (18). Eligible participants were U.S. residents, who were 18 years and older, and who either had one or more dogs and/or cats but no other types of pets (e.g., reptiles, fish, or other exotic pets). For those who had more than one dog and/or cat, we asked them to select the pet that they felt most attached to and assigned them to the corresponding survey based on their selection. In addition, to be eligible, participants must identify themselves as the primary caregiver of the pet chose for the study.

All data were collected anonymously and longitudinally across a total of six survey time points between June 2020 and December 2021. The initial survey was conducted in June 2020, and at the time of the survey, the data pertaining to the pre-pandemic period (February and April 2020) were also collected retrospectively. Follow-up surveys were conducted in September 2020, January, April, August, and December 2021. The study period was further divided into four different phases of the COVID-19 pandemic according to the U.S. Department of Defense coronavirus timeline.^1^ They were labeled as: (1) Pre-pandemic—February to April 2020, (2) Strict lockdown—June to December 2020, (3) Prolonged lockdown—January to April 2021, and (4) Re-opening—August to December 2021. Study participants were followed until they either reported a change in their pet ownership status or were lost-to-follow-up. We recruited additional participants since the January 2021 surveys in order to make up for the attrition from the earlier cohorts and to maintain the initially planned sample size (500 dog and 500 cat owners). These additional participants were combined with the earlier cohorts in the analysis.

### Survey questions

The survey began by collecting demographic information from participants, including age, gender, housing situation, and the composition of their household. The next section of the survey inquired about the frequency of HAI activities, which were grouped into categories such as playing or interacting with the pet, being away from the pet (e.g., not in the same room), dog walking, and the pet’s sleep location (e.g., in the bed, inside or outside the bedroom). The subsequent section focused on gathering information about the pet’s characteristics, including its sex, age, breed, weight, source of acquisition, and the length of ownership. Participants were asked about the presence of behavior problems and physiological changes in the month preceding the survey using a behavior checklist consisting of 19 binary behavior items.

These behavior items were further categorized into five groups: (1) Aggression (toward family members, unfamiliar people, household pets, unfamiliar pets), (2) Anxiety or fear (response toward noises, objects, people, animals, car rides, being left alone), (3) Repetitive behaviors (such as tail or light chasing, which applies to dogs only, and excessive licking), (4) House-soiling (inappropriate urination or defecation), and (5) Physiological changes (changes in appetite and sleep patterns). Additionally, two surveys examined two specific behavioral problems in dogs: aggression toward family members and anxiety related to being left alone. For cats, it explored urine marking on vertical surfaces. These specific problems were known to be correlated with environmental or routine changes, such as transitions before and after strict lockdown, and shifts from prolonged lockdown to reopening phases (19–21). Further details on the behavior checklist can be found in Supplementary Tables S1, S2.

### Statistical analysis

IBM SPSS Statistics (Version 29. Armonk, NY: IBM Corp.) was used for data analyses. All the analyses were done separately for dogs and cats. Statistical significance was set at *p* < 0.05. Bonferroni adjustment was applied for pairwise comparisons.

To assess whether occurrence of behavior problems varied across different phases of COVID pandemic (hypothesis 1), we used generalized estimating equations (GEE) with logistic link function and binomial distribution (i.e., logistic regression). Each behavior category was modeled as the response variable separately with phase, dummy coded, as the independent variable.

We investigated whether HAI activities varied across different COVID phases (hypothesis 2) using GEE—multinomial logistic regression, in which each HAI activity was modeled as the response variable with COVID phase as the independent variable. For hypothesis 3, we modeled the association between HAI (independent variables) and behavior problems (response variables) during COVID using GEE—logistic regression. In addition, we also investigated pet signalment (e.g., age and sex of pet, length of ownership, and size of dog), house type (apartment, house with or without fenced yard, and others), and whether there were young children (<6 years old) in the household as potential confounders. Significant covariates and phase were adjusted for in the final GEE—logistic regression model. When applicable, adjusted odds ratio (OR) and 95% confidence interval (CI) were reported.

## Results

### Study population

A total of 3,278 participants completed one to six surveys during the study period, with the overall response rates for at least two surveys of 43 and 43% for dog and cat surveys, respectively. The initial cohort established in June 2020 comprised 37% (*n* = 1,224) of the total study population, whereas additional participants were recruited in January (*n* = 938), April (*n* = 164), August (*n* = 527), and December 2021 (*n* = 425). Among participants, 1,090 (33%) had dog only, 1,009 (31%) had cat only, and 1,179 (36%) had both dog and cat in the household at the time of the first survey. The most frequent age group for dogs was 3–7 years old (43%) and for cats was 3–6 years old (35%). The majority of dogs were obtained from breeders (34%) or shelters (38%), whereas the top two acquisition sources for cats were shelter/rescue (46%) and birth of a family pet (21%). Other demographic information of the participants and the characteristics of the pets are summarized in Table 1.

**Table 1:** Demographic information about the study population of dog and cat owners in North America (*N* = 3,278).

Primary caregivers			
**Gender**			
Female	1,667(51%)		
Male	1,578 (48%)		
Not determined	26 (0.08%)		
**Household pets**			
Dog only	1,090 (33%)		
Cat only	1,009 (31%)		
Both dog and cat	1,179 (36%)		

Dog		Cat	
**Sex**			
Female intact	391 (24%)	Female intact	228 (14%)
Female spayed	474 (29%)	Female spayed	559 (34%)
Male intact	272 (17%)	Male intact	283 (17%)
Male neutered	493 (30%)	Male neutered	578 (35%)
**Current age**			
<6 M	24 (1.7%)	<6 M	31 (2.1%)
6 M—2 years	334 (23%)	6 M–2 years	423 (29%)
3–7 years	620 (43%)	3–6 years	515 (35%)
8–12 years	360 (25%)	7–10 years	244 (17%)
13–15 years	86 (5.9%)	11–14 years	183 (12%)
≥16 years	28 (1.9%)	≥15 years	79 (5.4%)
**Source**			
Birth of family pet	287 (18%)	Birth of family pet	330 (21%)
Breeder	532 (34%)	Breeder	218 (14%)
Shelter/rescue	587 (38%)	Shelter/rescue	727(46%)
Other	154 (9.9%)	Other	318 (20%)
**Age at acquisition**			
<2 M	353 (24%)	<2 M	445 (27%)
2–5 M	572 (38%)	2–6 M	536 (33%)
6 M—1 year	323 (22%)	6 M—1 year	309 (19%)
2–7 years	208 (14%)	1–3 years	262 (16%)
>7 years	36 (2.4%)	3–7 years	68 (4.1%)
		>7 years	27 (1.6%)
**Length of ownership**			
<1 year	99 (8.8%)	<1 year	140 (12%)
1–5 years	541 (48%)	1–5 years	783 (64%)
>5 years	480 (43%)	>5 years	293 (24%)

### Trend of behavior problems through COVID phases

**Figure 1:**
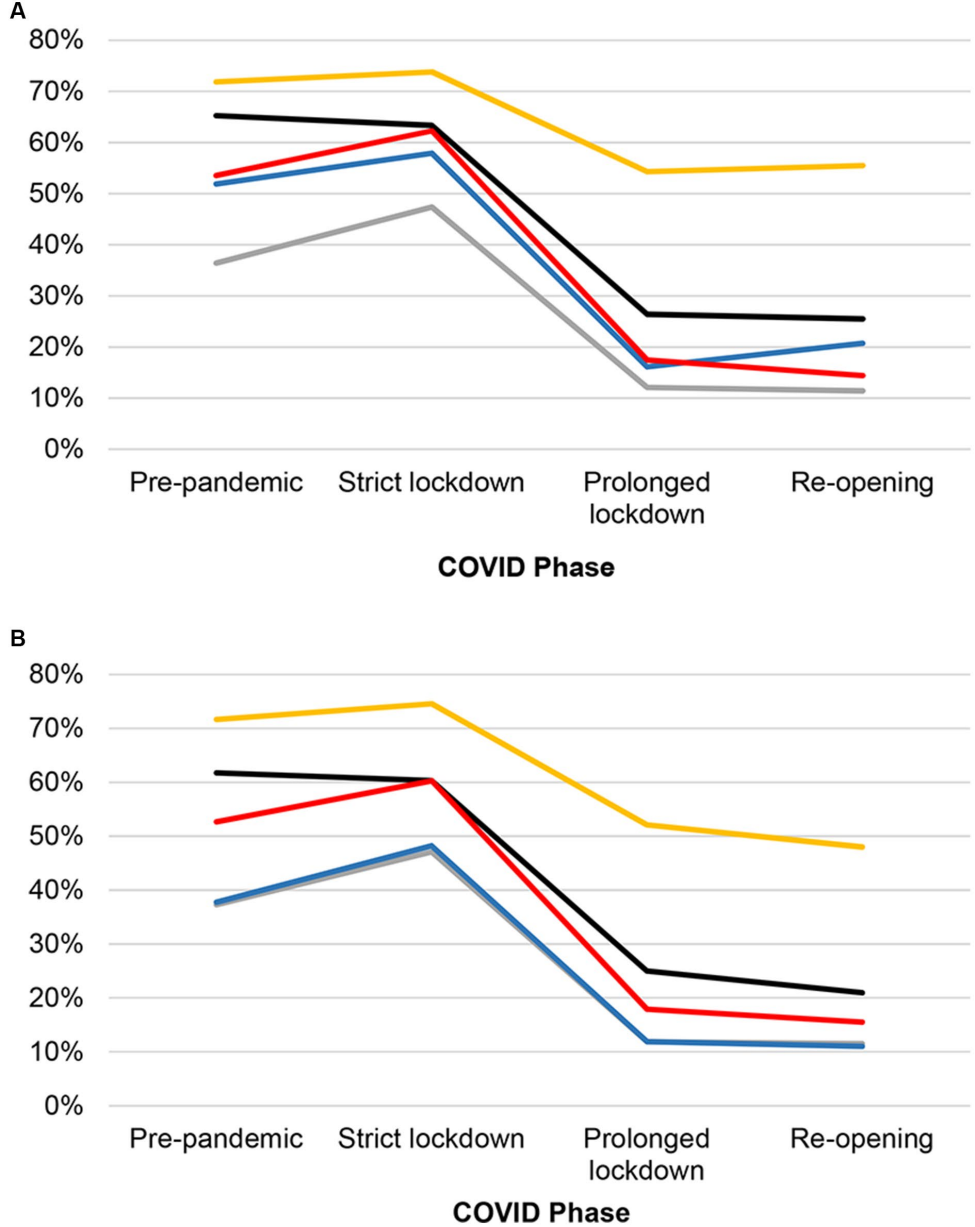
Temporal changes in the proportion of behavior problems in **(A)** dogs and **(B)** cats over different COVID phases between February 2020 and December 2021. Black line: Aggression; Yellow line: Anxiety or fear; Gray line: House-soiling; Blue line: Repetitive behaviors; Red line: Physiological changes.

The results showed significant differences in the occurrence of all behavior categories across COVID phases in both dogs and cats (all *p*’s < 0.001), supporting hypothesis 1. In both dogs and cats, most behavior categories followed a similar pattern: a slight change from Pre-pandemic to Strict lockdown phase, followed by a substantial decrease in Prolonged lockdown phase (Figure 1). The occurrence of behavior problems remained lower level in Re-opening compared to Pre-pandemic phase. Notably, despite statistical significance across COVID phases, the occurrence of anxiety or fear in both species showed a subtler declining from Strict lockdown to Prolonged lockdown compared to other behaviors in both species.

**Figure 2:**
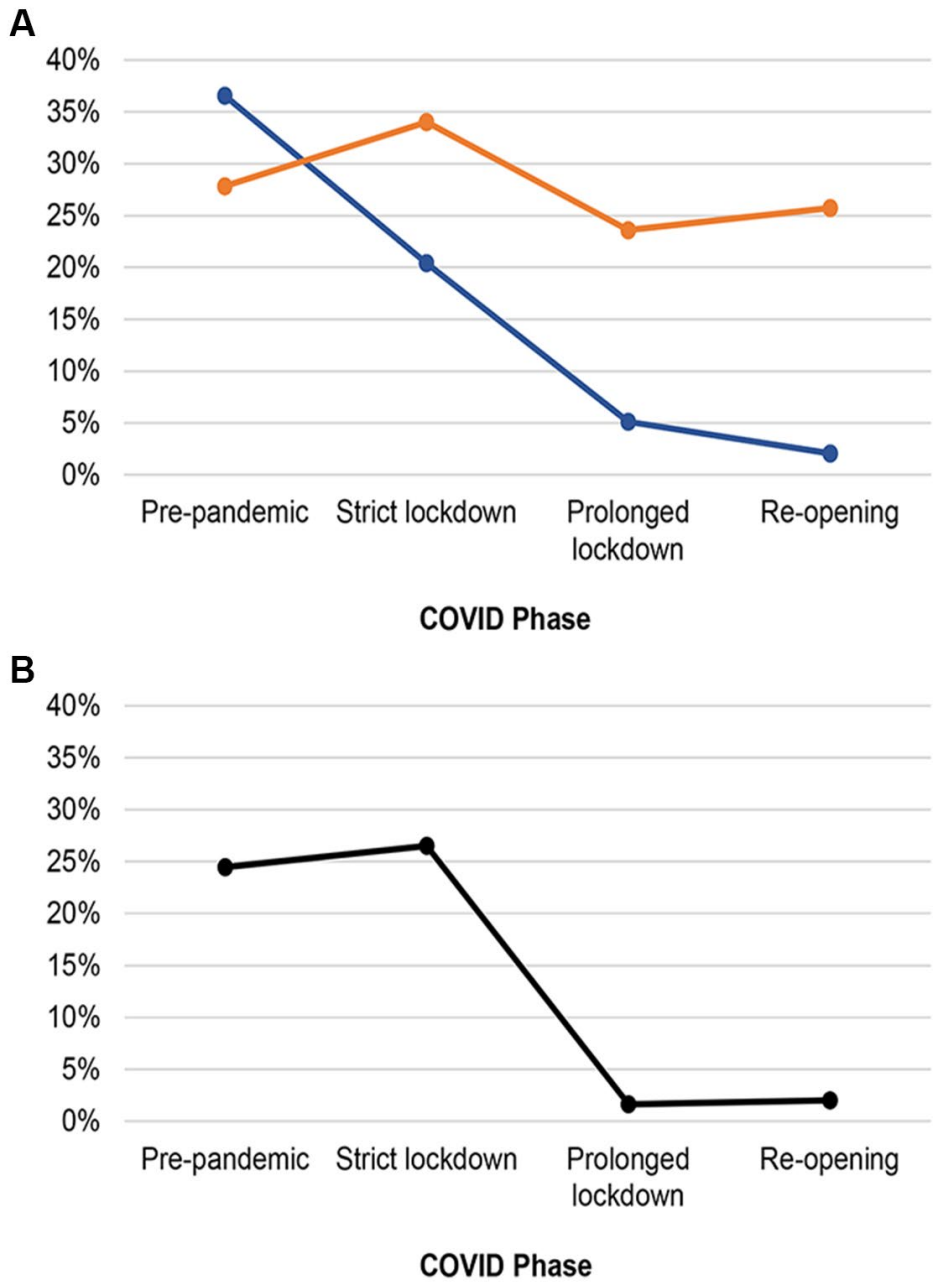
Temporal changes in the proportion of specific behavior problems in **(A)** dogs and **(B)** cats over different COVID phases between February 2020 and December 2021. Blue line: Aggression toward family members; Orange line: Anxiety of being left alone; Black line: Urine marking at vertical surfaces.

Figure 2A illustrated that dogs were more likely to exhibit aggression toward family members during Pre-pandemic compared to Strict lockdown (OR = 2.2, 95% CI 1.6–3.1) and during Strict lockdown compared to Prolonged lockdown (OR = 4.7, 95% CI 3.2–7.0). Dogs were also more likely to exhibit aggression toward family members during Prolonged lockdown compared to the Re-opening phase (OR = 2.6, 95% CI 1.3–4.9). The occurrence of anxiety related to being left alone in dogs also differed across COVID phases (*p* < 0.001), although overall differences between phases were smaller than the differences in aggression toward family members (see Figure 2A). Specifically, dogs were slightly more likely to show anxiety related to being left alone during Strict lockdown compared to Pre-pandemic (OR = 1.3, 95% CI 1.0–1.8) and Prolonged lockdown phases (OR = 1.7, 95% CI 1.3–2.1). There were no differences between Pre-pandemic, Prolonged lockdown, and Re-opening phases.

In Figure 2B, it was observed that cats were more likely to exhibit urine marking behavior during Strict lockdown when compared to Prolonged lockdown (OR = 22, 95% CI 12–39). However, there were no significant differences in urine marking behavior between Pre-Pandemic and Strict lockdown (OR = 0.90, 95% CI 0.65–1.2) and between Prolonged lockdown and Re-opening (OR = 0.81, 95% CI 0.35–1.9).

### Trend of HAI through COVID phases

**Figure 3:**
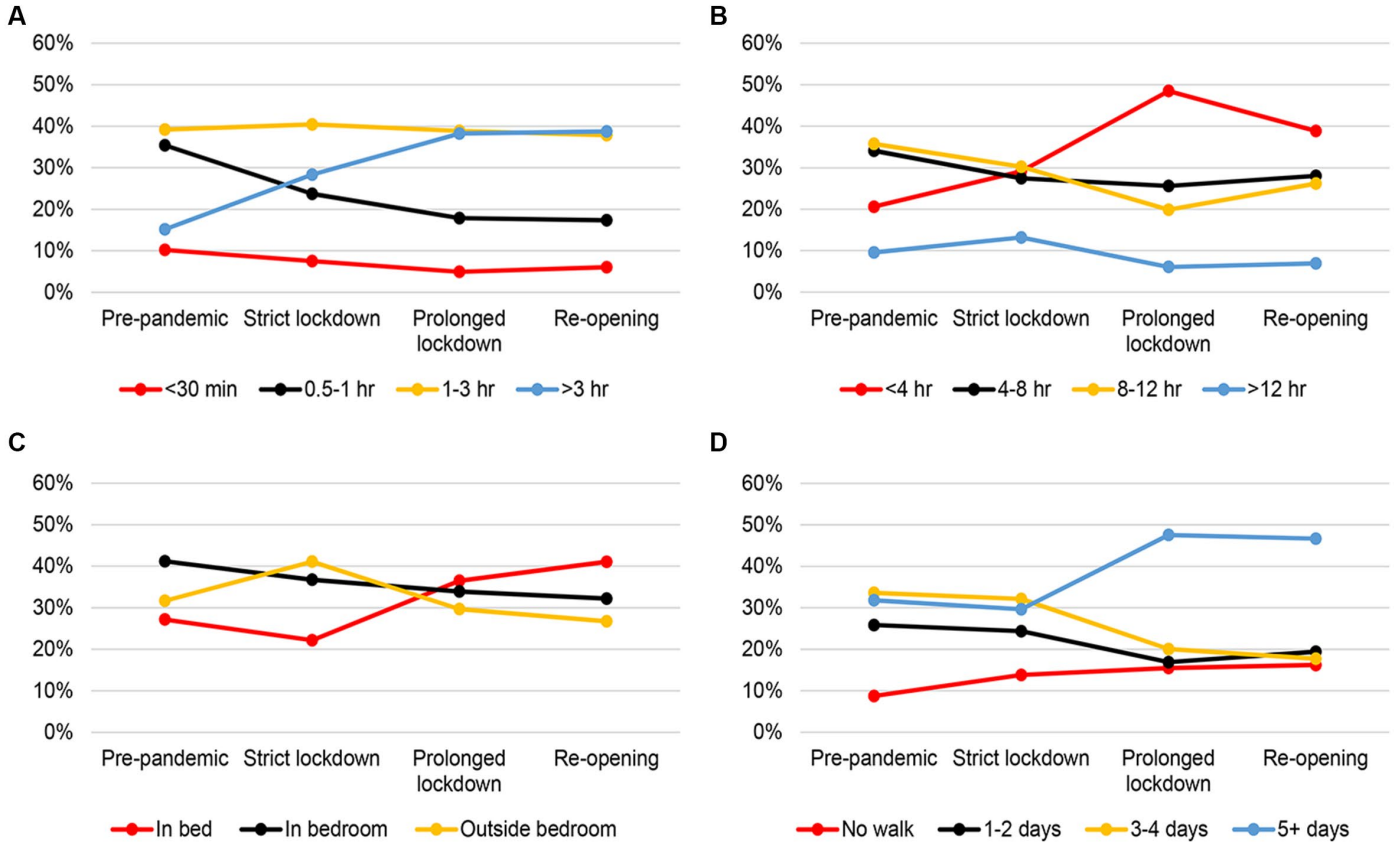
Trends in the distribution of different owner-dog interaction activities over different COVID phases between February 2020 and December 2021. **(A)** Amount of time playing or interacting with the dog, **(B)** amount of time being away from the dog, **(C)** sleep location of the dog, and **(D)** frequency of dog walking.

In both dogs and cats all HAI activities varied across COVID phases (all *p*’s < 0.001), which supported hypothesis 2. Figure 3 illustrated that the proportion of owners spending >3 h per day interacting with their dogs continued to increase from Pre-pandemic to Prolonged lockdown phase. Conversely, a decreasing trend was observed in the proportion of owners spending 0.5–1 h/day interacting with their dogs during the same time period. Among the four groups related to time being away from the dog, the <4 h/day group showed the greatest variation compared to the other groups. The proportion of owners being away from their dogs for <4 h/day increased from 21% during Pre-pandemic to 49% in Prolonged lockdown, and then decreased to 39% during Re-opening phase.

For sleep location, it was observed that the proportion of dogs slept in the owner’s bed doubled after Strict lockdown, while the other two groups showed a declining trend. In the case of dog walking, the proportion of owners who did not walk their dogs continued to gradually increase from Pre-pandemic phase. Among dog owners who walked their dogs, a shift in the pattern was noticed from Strict lockdown to Prolonged lockdown phase. During this time, the proportion of owners who walked their dogs for ≥5 days/week increased from 30% during Strict lockdown to nearly 50% in Prolonged lockdown and Re-opening phases, while the proportion of owners who walked their dogs for <5 days/week decreased.

**Figure 4:**
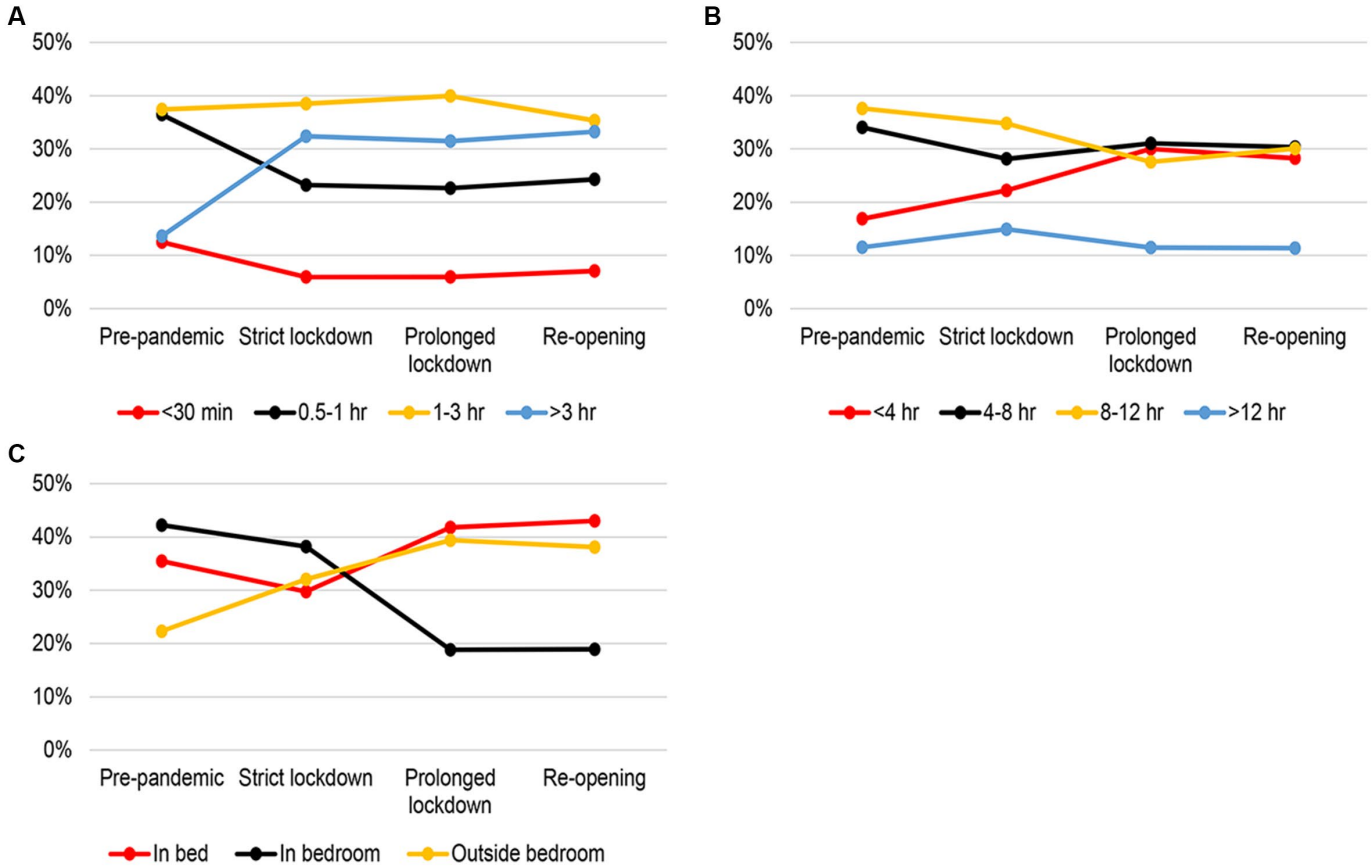
Trends in the distribution of different owner-cat interaction activities over different COVID phases between February 2020 and December 2021. **(A)** Amount of time playing or interacting with the cat, **(B)** amount of time being away from the cat, and **(C)** sleep location of the cat.

Figure 4 illustrated that the proportion of cat owners spending >3 h/day interacting with their cats increased from Pre-pandemic to Strict lockdown phase. In contrast, the proportion of owners spending 0.5–1 h/day decreased during the same time period. All proportions remained relatively stable from Prolonged lockdown phase until Re-opening phase. As expected, the proportion of owners being away from their cats for <4 h/day continued to increase from Pre-pandemic phase until Prolonged lockdown, while the proportion of owners being away for 8–12 h/day decreased over the same phases. The proportion of owners who were away for >12 h/ day did not change over the COVID-19 phases. Interestingly, the proportion of cats sleeping in the owner’s bedroom showed a different pattern compared to the other two sleep location groups. The proportion of cats sleeping in the bedroom decreased from Pre-pandemic (42%) to 19% in Prolonged lockdown and Re-opening phases, while the other two groups showed an increasing trend over the same period.

### Association between HAI and behavior problems during COVID

**Figure 5:**
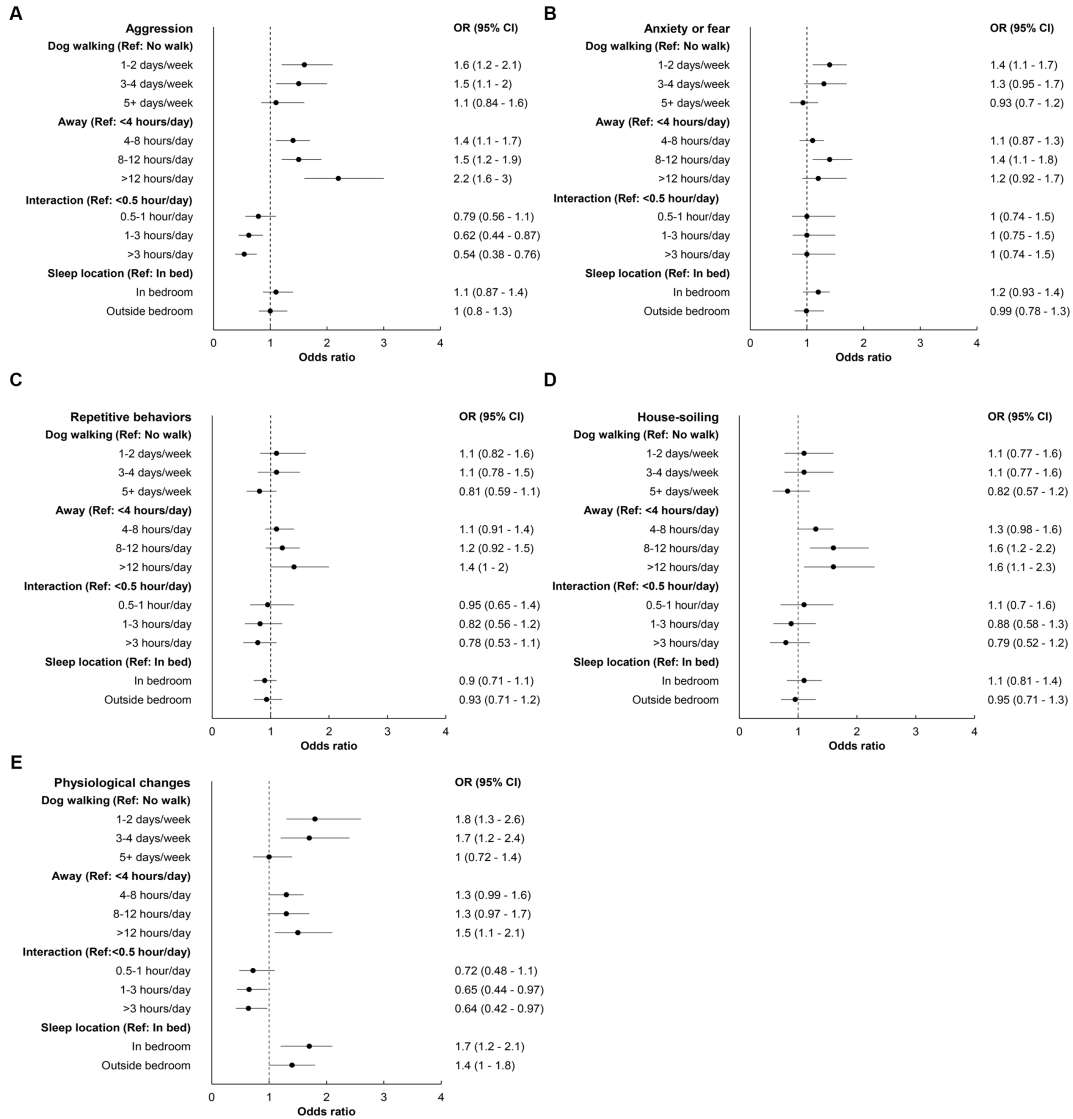
Forest plots of odds ratio (OR) and 95% confidence interval (CI) depicting the association between human-animal interaction activities and different behavior problems in dogs: **(A)** aggression, **(B)** anxiety or fear, **(C)** repetitive behaviors, **(D)** house-soiling, and **(E)** physiological changes. Dots are ORs and the whiskers are 95% CIs. The dashed vertical line intercepting 1 represents the null value of OR (i.e., no association).

Lastly, we examined the association between HAI and behavior problems through the COVID phases (hypothesis 3). Figure 5 indicated that, overall, HAI activities showed the strongest association with aggression and aggression toward family members in dogs. For aggression and aggression toward family members, all HAI showed strong associations except for dog’s sleep location. The associations between HAI and all other behavior categories were relatively weaker. However, being away consistently showed a positive association (i.e., being away longer associated with higher odds of behavior issues), and interaction an inverse association (i.e., spending more time interacting with the dog associated with lower odds of behavior issues) across different behavior categories. Moreover, owners who walked their dogs occasionally were more likely to report behavior issues than owners who did not walk their dogs and owners who consistently walked their dogs (i.e., ≥5 days/week). Dog’s sleep location only associated with physiological changes. Anxiety of being left alone had weak associations with all HAI activities.

**Figure 6:**
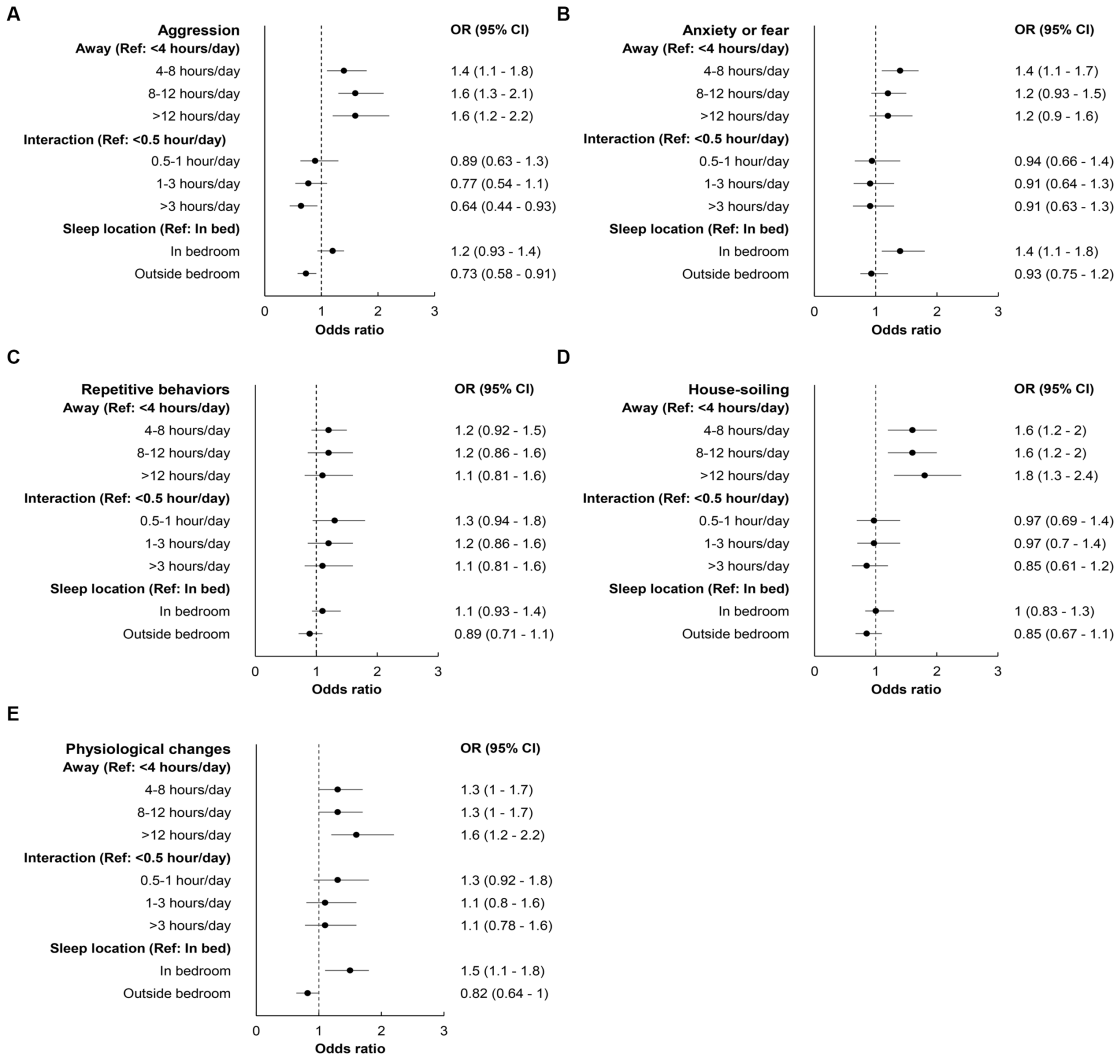
Forest plots of odds ratio (OR) and 95% confidence interval (CI) depicting the association between human-animal interaction activities and different behavior problems in cats: **(A)** aggression, **(B)** anxiety or fear, **(C)** repetitive behaviors, **(D)** house-soiling, and **(E)** physiological changes. Dots are ORs and the whiskers are 95% CIs. The dashed vertical line intercepting 1 represents the null value of OR (i.e., no association).

Similar to dogs, HAI activities showed an overall strongest association with aggression in cats (Figure 6). A consistent positive association between being away and behavior problems and inverse association between interaction and behavior problems were also observed in cats. Different from dogs, cat’s sleep location showed a stronger association with aggression, anxiety or fear, and physiological changes, with sleeping outside bedroom tended to have the lowest odds. Urine marking had weak associations with all HAI activities.

## Discussion

The onset of the COVID-19 pandemic compelled us to adapt to abrupt changes in our lifestyles. Questions regarding its potential influences on pet behavior began to emerge. These included the direct consequences of owners spending more time at home, reduced physical interaction with others, and other related lifestyle changes (6, 8). In response, we conducted a survey-based longitudinal investigation to characterize the dynamics of pet behavior, human-animal interaction activities, and their associations during the course of the COVID pandemic.

This study possessed several distinctive attributes in comparison to previous studies. Firstly, it was a longitudinal study covering a longer timeframe from February 2020 to December 2021, incorporating retrospective baseline data from February and April 2020. While previous studies had primarily focused on the Strict lockdown phase, this study continued to track changes after it by including the Prolonged lockdown and Re-opening phases. Second, in contrast to earlier research, the present study directly quantified HAI (Human-Animal Interaction) activities by inquiring about the amount of time owners spent interacting with their pets or being away from them on a daily basis, rather than inferring such activities based solely on the reported duration of staying home. Lastly, to improve the representativeness of the study population of general dog and cat owners in the United States, we employed an online crowdsourcing platform to recruit a large and diverse sample. This approach also ensured the acquisition of high-quality data (22), as opposed to relying on potentially biased sampling through social media platforms dedicated to pet communities.

The study findings supported our first hypothesis, which stated that behavior problems and physiological changes in dogs and cats would vary across the four COVID phases.

However, when compared to Pre-pandemic phase, there was only a marginal increase in the occurrence of behavior problems in both species during Strict lockdown. While this finding concurred with some of the previous studies, the inconsistency among studies is noteworthy.

Our finding of only subtle increases during Strict lockdown might be attributed to the retrospective data collection for Pre-pandemic phase (i.e., data for February and April 2020 were collected in June 2020). However, this was inevitable, as an outbreak investigation, including this study, would be initiated only after the confirmation of an outbreak. Nonetheless, it is worth noting that the occurrence of behavior problems during our Pre-pandemic phase (73%) was slightly lower than the reported prevalence in previous studies for dogs with at least one behavior problem (85–86%) (23, 24). The prevalence for cats (72%) fell within the range reported in previous studies (16–76%) (25–28). However, it is important to acknowledge that the behavior categories used in each study were different, which likely contributed to the discrepancies among the studies (23). Another possible explanation is that women were often overrepresented in pet-related surveys (9, 29), including those conducted in previous studies. In our study, the gender distribution was representative of the general U.S. population with 48% men and 51% women.

When compared to other behavior problems, anxiety or fear-related problems were the most common in both dogs and cats. They exhibited relatively modest fluctuations across the different phases. This finding corresponded with the observation of only minor variations in anxiety when dogs were left alone across the COVID phases. Anxiety or fear-related problems were also the most commonly reported behavior problems in pet dogs (32–44%) prior to the COVID-19 pandemic (23, 30). This suggests that anxiety or fear-related problems likely remain prevalent and are most acknowledged by owners. Additionally, it is known that anxiety or fear-related problems have a strong genetic contribution (30), which might lead to outcomes less influenced by external factors such as changes across the COVID phases.

Notably, the occurrence of aggression toward family members began to decline starting from the Pre-pandemic phase. This contrasted with the trends of other behavior problems, which only began to decline after Strict lockdown. This divergence is particularly noteworthy when compared with other studies reporting an increase in dog-bites involving children (11, 12). However, it is important to note that those studies focused on children visiting emergency departments, whereas our study involved reports from general pet owners, making direct comparisons less applicable.

The declining trends in aggression and other behavior problems during the pandemic also correlated with the earlier finding of a stronger owner-pet relationship among our study participants (31). It is known that owners might experience caregiver burden when their pets had behavior problems (32). Thus, less behavior problems might result in a stronger bond or vice versa. Despite these discrepancies, the overarching trajectory of most behavior problems, including feline urine marking, indicated a sustained deviation from the baseline (i.e., the Pre-pandemic phase). This departure persisted, with the occurrence of behavior problems continuously to decline as the COVID phases transitioned from the Strict lockdown to the Re-opening phase.

When exploring HAI activities across the COVID phases, the duration owners spent engaging with their pets surged during the Strict and Prolonged lockdown phases. The overall findings of HAI activities supported hypothesis 2. Intriguingly, notable disparities in HAI activities were observed between dog and cat owners after Strict lockdown. Among dog owners, overall interactions with dogs increased, time being away from dogs decreased, instances of dogs sharing the owner’s bed increased, and there were more frequent walks. These trends aligned with recent reports from other studies highlighting the values of pet-friendly workplaces (PFWs) (33, 34). The studies illustrated that PFWs became important for many companies as it helped with employee recruitment and retention. Our study and those PFW-related studies reflect the public trend of spending more time with their pets during lockdown carrying over as a new lifestyle.

Compared to dog owners and their dogs, the HAI activities between cat owners and their cats exhibited different temporal patterns across the COVID phases. For instance, the proportion of cats sleeping in the bedroom declined substantially from Strict lockdown to Prolonged lockdown and stayed at a lower level during Re-opening compared with Pre-pandemic. Correspondingly, the proportion of cats sleeping outside the bedroom gradually increased from Pre-pandemic till Prolonged lockdown.

Considering that cats are typically not taken for a walk and are kept indoors, this behavior trajectory might suggest a coping style for cats in response to altered owner schedules while upholding territorial boundaries and routines (6, 35).

Overall, the associations between HAI activities and behavior problems were stronger in dogs than cats. However, the two species shared a few similarities. For example, HAI activities showed the strongest association with aggression, physiological changes, followed by house-soiling in both dogs and cats. In addition, for both dogs and cats, anxiety or fear behavior problems showed either no or very weak association with HAI activities. Given that anxiety is a chronic condition known to require a multimodal approach, especially in severe cases (36), this finding aligns with existing knowledge.

An interesting finding emerged, underscoring a non-linear association between the frequency of dog walking and behavior problems. The results indicated that both the “no walk” and “frequent dog walking” groups (i.e., ≥5 days/week) exhibited fewer occurrences of behavior problems compared to the “occasional dog walking” groups (i.e., 1–4 days/week). This insight implies that not only the quantity of exercise, but also adherence to a consistent schedule, plays a pivotal role in mitigating behavior problems in dogs. This observation aligns with the principle that predictability in routine fosters coping mechanisms (37).

Interactions with dogs showed a consistent inverse association with the occurrence of behavior problems, while being away from dogs showed a consistent positive association. These findings suggest a clear trend among dogs. It appears that when there is an increase in HAI activities (e.g., owners interact with their dogs or being closely with them), a virtuous cycle is set in motion. This cycle involves more interactions leading to fewer behavior problems, ultimately strengthening the bond between humans and their canine companions (38).

In contrast, while interactions with cats showed an inverse association with the majority of behavior problems, being away from cats did not consistently exhibit a positive association, as observed in dogs. Additionally, the association between sleep location and the occurrence of behavior problems also exhibited species differences. Among cats that slept outside the bedroom, the occurrence of aggression, anxiety or fear, as well as physiological behavior changes, was the lowest (39). Conversely, dogs that slept outside the bedroom either showed no association or exhibited a weak one with an increased occurrence of physiological changes. These findings indicate a notable distinction between dogs and cats in their responses to the changes during the pandemic. When compared to Pre-pandemic, some cats displayed an increased desire for attention from their owners during the pandemic. On the other hand, other cats exhibited a preference for avoiding such interactions. This divergence underscores the species differences in social interactions between dogs and cats (8, 40). Given the fewer studies on cat behaviors (3, 6–8, 10, 38) compared to dogs (3–6, 8–9, 11–13, 16–17) during the COVID pandemic, these findings emphasize the importance of considering species-specific factors in future studies of pet cats.

While acknowledging certain limitations, it is crucial to emphasize that the assessment of behavior problems relied on self-reported data collected via surveys. Furthermore, we did not assess the severity of a behavior problem, but instead gathered dichotomous values (yes/no) for each behavior. The fact that some participants withdrew from the study due to giving up their pets could potentially impact the subsequent data analysis. This non-random attrition might introduce selection bias into the results, possibly leading to an underestimate of the occurrence of behavior issues and an overestimate of certain HAI activities. Another concern was about the validity of the baseline data (February and April 2020) collected in the initial survey in June 2020. Considering the retrospective nature of the data, it is important to acknowledge the potential for recall bias. Further studies involving direct observation, such as using video recording or employing wearable devices (e.g., activity monitors on both humans and animals), may address some of the limitations of the study and provide stronger evidence to support the development of policies and best practices to mitigate impact of an adverse effect on human-animal relationships.

Nevertheless, the study’s primary strength resides in being the first longitudinal investigation to encompass the pandemic’s inception and its subsequent phases. The study findings provide invaluable insights into public concerns regarding pet welfare, shedding light on the species-specific differences in HAI dynamics during significant environmental changes, such as a pandemic. The finding of species differences can facilitate the implementation of interventions for the unique needs of each species through human-animal interactions to mitigate the occurrence of behavioral problems and enhance human-animal relationships.

## Data availability statement

The original contributions presented in the study are included in the article/Supplementary material, further inquiries can be directed to the corresponding author.

## Ethics statement

The studies involving humans were approved by the Purdue University Human Research Protection Program Institutional Review Board. The studies were conducted in accordance with the local legislation and institutional requirements. Written informed consent for participation was not required from the participants or the participants’ legal guardians/next of kin in accordance with the national legislation and institutional requirements.

## Author contributions

H-YW: Conceptualization, Data curation, Formal analysis, Funding acquisition, Investigation, Methodology, Validation, Visualization, Writing – review & editing. NO: Conceptualization, Funding acquisition, Investigation, Methodology, Project administration, Resources, Validation, Writing – original draft, Writing – review & editing.

## Footnotes

^1^ https://www.defense.gov/Spotlights/Coronavirus-DOD-Response/Timeline/
