# Supplementary material for: The impact of COVID-19 pandemic on pet behavior and human-animal interaction: a longitudinal survey-based study in the United States
Source: Front Vet Sci. 2023 Nov 28;10:1291703. doi: 10.3389/fvets.2023.1291703 (PMC10716918; doi:10.3389/fvets.2023.1291703)
Supplement: Supplementary file 1 [file Table_1.DOCX]

Q. **Please share behavior profile regarding this dog**Please check all behavior concerns regarding this dog last month.

Table S1: Behavior checklist for dogs

| Behavior concerns | Occurrence |
| --- | --- |
| Aggression to household members |  |
| Aggression to unfamiliar humans |  |
| Aggression to housemate dogs |  |
| Aggression to unfamiliar dogs |  |
| Fear/anxiety to loud noises |  |
| Fear/anxiety to objects (e.g., furniture) |  |
| Fear/anxiety to people |  |
| Fear/anxiety to animals |  |
| Fear/anxiety to new places, and/or car rides |  |
| Fear/anxiety to be left alone |  |
| Excessive licking or grooming |  |
| Tail chasing or biting |  |
| Chasing or biting light/shadow |  |
| House soiling (urine) |  |
| House soiling (defecation) |  |
| Decreased appetite |  |
| Increased appetite |  |
| Decreased total sleep time |  |
| Increased total sleep time |  |

Q. **Please share behavior profile regarding this cat**Please check all behavior concerns regarding this cat last month.

Table S2: Behavior checklist for cats

| Behavior concerns | Occurrence |
| --- | --- |
| Aggression to household members |  |
| Aggression to unfamiliar humans |  |
| Aggression to housemate cats |  |
| Aggression to unfamiliar cats |  |
| Fear/anxiety to loud noises |  |
| Fear/anxiety to objects (e.g., furniture) |  |
| Fear/anxiety to people |  |
| Fear/anxiety to animals |  |
| Fear/anxiety to new places, and/or car rides |  |
| Fear/anxiety to be left alone |  |
| Excessive licking or grooming |  |
| Tail chasing or biting |  |
| Urination at vertical surface |  |
| Urination at horizontal surface (out of litter box) |  |
| Defecation out of litter box |  |
| Decreased appetite |  |
| Increased appetite |  |
| Decreased total sleep time |  |
| Increased total sleep time |  |
