# Supplementary material for: The impact of COVID-19 pandemic on pet behavior and human-animal interaction: a longitudinal survey-based study in the United States
Source: Front Vet Sci. 2023 Nov 28;10:1291703. doi: 10.3389/fvets.2023.1291703 (PMC10716918; doi:10.3389/fvets.2023.1291703)
Supplement: Supplementary file 2 [file Data_Sheet_1.PDF]

**Primary caregivers**			
Gender			
Female	1667(51%)		
Male	1578 (48%)		
Not determined	26 (0.08%)		
Household pets			
Dog only	1090 (33%)		
Cat only	1009 (31%)		
Both dog and cat	1179 (36%)		
**Dog**		**Cat**	
Sex			
Female intact	391 (24%)	Female intact	228 (14%)
Female spayed	474 (29%)	Female spayed	559 (34%)
Male intact	272 (17%)	Male intact	283 (17%)
Male neutered	493 (30%)	Male neutered	578 (35%)
Current Age			
<6M	24 (1.7%)	<6M	31 (2.1%)
6M – 2yrs	334 (23%)	6M – 2yrs	423 (29%)
3 – 7 yrs	620 (43%)	3 – 6 yrs	515 (35%)
8 – 12 yrs	360 (25%)	7 – 10yrs	244 (17%)
13 – 15 yrs	86 (5.9%)	11 – 14 yrs	183 (12%)
≥16 yrs	28 (1.9%)	≥15 yrs	79 (5.4%)
Source			
Birth of family pet	287 (18%)	Birth of family pet	330 (21%)
Breeder	532 (34%)	Breeder	218 (14%)
Shelter/rescue	587 (38%)	Shelter/rescue	727(46%)
Other	154 (9.9%)	Other	318 (20%)
Age at acquisition			
<2 M	353 (24%)	<2 M	445 (27%)
2 – 5 M	572 (38%)	2 – 6 M	536 (33%)
6M – 1 yr	323 (22%)	6M – 1 yr	309 (19%)
2 – 7 yrs	208 (14%)	1 – 3 yrs	262 (16%)
>7yrs	36 (2.4%)	3 – 7 yrs	68 (4.1%)
		>7yrs	27 (1.6%)
Length of ownership			
<1yr	99 (8.8%)	<1yr	140 (12%)
1 – 5yrs	541 (48%)	1 – 5yrs	783 (64%)
>5yrs	480 (43%)	>5yrs	293 (24%)
